# Case Report: Cadonilimab-related toxic epidermal necrolysis-like reactions successfully treated with supplemental Adalimumab

**DOI:** 10.3389/fimmu.2023.1188523

**Published:** 2023-08-03

**Authors:** Peng-Yu Chen, Zi-Yun Li, Sui-Qing Cai

**Affiliations:** Department of Dermatology, Second Affiliated Hospital of Zhejiang University School of Medicine, Hangzhou, China

**Keywords:** case report, Cadonilimab/AK104, toxic epidermal necrolysis, Adalimumab, immune-related adverse events, immune checkpoint inhibitor

## Abstract

Cadonilimab is the first bi-specific antibody approved for certain malignancies in June 2022, which has a modified Fc structure to reduce immune-related adverse events. To date, no reports have described Cadonilimab-related toxic epidermal necrolysis (TEN). Here, we report the first case of TEN-like reactions occurring during the treatment of hepatocellular carcinoma with Cadonilimab in combination with Lenvatinib and transarterial chemoembolization, successfully treated with supplemental Adalimumab. We confirmed Cadonilimab as the culprit and observed significant improvement in the patient’s condition following Adalimumab treatment. The case emphasizes the potential risk of Cadonilimab inducing TEN, and suggests that supplemental Adalimumab could be a favorable option for treating refractory Cadonilimab-related TEN.

## Introduction

Cadonilimab is the first bi-specific antibody with a crystallizable fragment (Fc) effector null backbone which targets both programmed death 1 (PD-1) and cytotoxic T-lymphocyte-associated protein 4 (CTLA-4), and was approved for the treatment of certain malignancies in China in June 2022 (1, 2). While immune checkpoint inhibitors (ICIs) have supplemented cancer therapy, they can also trigger immune-related adverse events (irAEs), including rare high-grade skin adverse events such as Stevens-Johnson syndrome (SJS) and toxic epidermal necrolysis (TEN). To date, no reports have described Cadonilimab-related TEN. This report aims to present a case of TEN-like reactions that occurred during the treatment of hepatocellular carcinoma (HCC) with Cadonilimab in combination with Lenvatinib and transarterial chemoembolization (TACE). We confirmed that Cadonilimab was the culprit and successfully treated the patient with routine treatment plus Adalimumab.

## Case description

**Figure 1:**
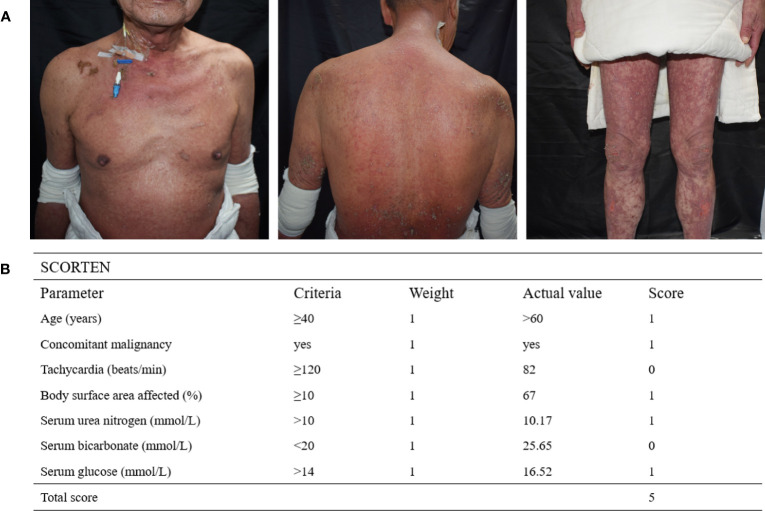
**(A)** The patient’s severely detached epidermal site was covered with dressings, and diffuse erythematous patches with erosions, ruptured loose bulla, and multisite positive Nikolsky’s sign were observed. **(B)** SCORTEN is assessed based on seven independent parameters within the first 24 hours of admission, with a total score of 5 indicating an estimated mortality rate of 90%.

A male patient in his 60s was admitted to our hospital after experiencing widespread erythema with blister formation, skin pain, and mucosal detachment for 5 days (**Figure 1A** **)** and showed no improvement after receiving methylprednisolone (4 mg/kg/d) and intravenous immunoglobulin (IVIG) (0.4 g/kg/d) treatment for 3 days. The patient had previously undergone TACE 35 days ago and subsequently received two courses of immunotherapy with Cadonilimab (10 mg/kg, administered 29 and 8 days prior to admission) and Lenvatinib (12 mg, once daily) for unresectable HCC. After considering the medication history and clinical condition, particularly the presence of multisite positive Nikolsky’s sign, the diagnosis of TEN was strongly suspected. However, due to the atypical skin lesions and the patient’s refusal to undergo skin biopsy, the diagnosis was conservatively considered as TEN-like reactions. The Severity-of-Illness Score for TEN (SCORTEN (3)) of 5 was calculated based on objective parameters recorded by dermatologists experienced in managing TEN upon admission (**Figure 1B** **)**. The total area of detachment (including Nikolsky’s sign-positive areas) accounted for 67%.

**Figure 2:**
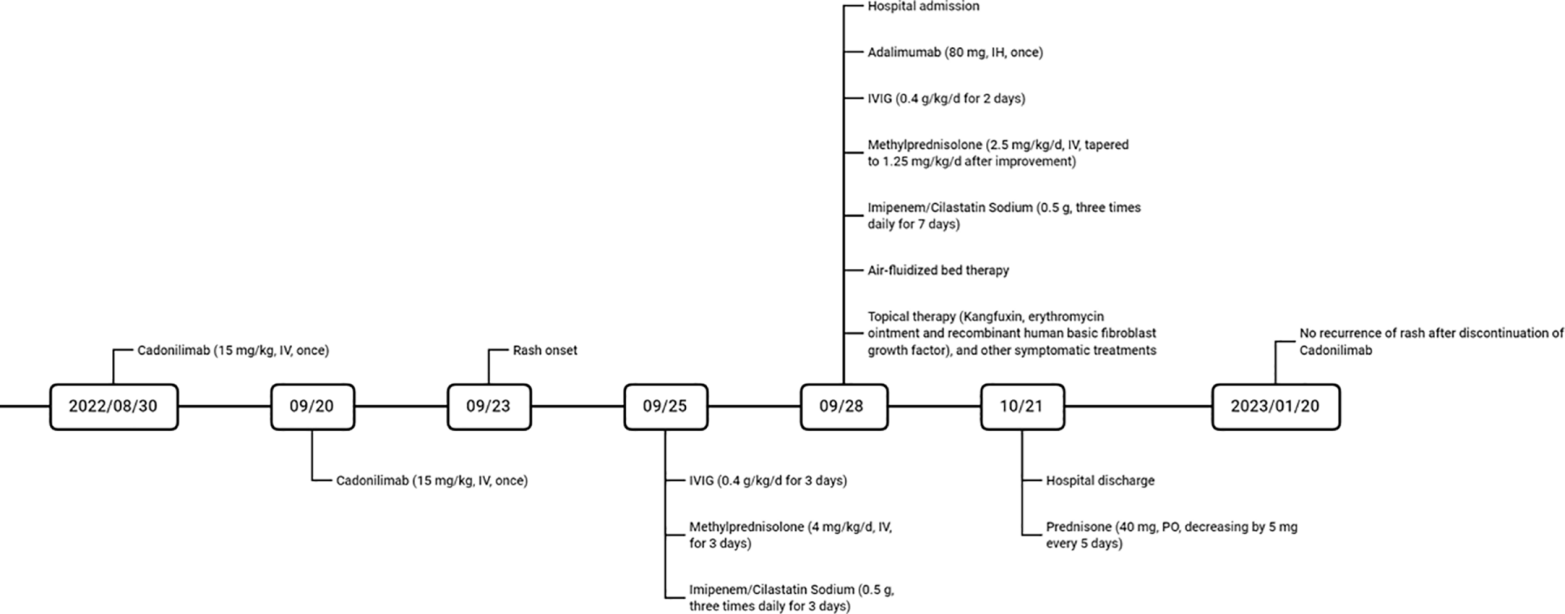
The timeline of medication history and treatments.

The treatment regimen included cessation of suspected drugs, administration of Adalimumab (80 mg, once), IVIG (0.4 g/kg/d, once daily for 5 consecutive days), methylprednisolone (2.5 mg/kg/d, tapered to 1.25 mg/kg/d after improvement, twice daily), and Imipenem/Cilastatin Sodium (0.5 g, three times daily), as well as air-fluidized bed therapy and topical therapy with Kangfuxin, erythromycin ointment, recombinant human basic fibroblast growth factor, and other symptomatic treatments (**Figure 2**). After a 23-day hospitalization, the patient’s erythema and erosions had nearly resolved, leaving pigmentation. After discharge, the patient took Prednisone (40 mg, decreasing by 5 mg every 5 days) and received TACE for unresectable HCC at the oncology hospital. Follow-up at 3 months showed no new eruptions.

## Discussion

Although the patient was diagnosed with TEN-like reactions, the nature of the underlying pathogenesis suggests drug-induced TEN. The patient had only been administered TACE-related drugs, hepatoprotective agents, celecoxib, Cadonilimab, and Lenvatinib within six months before onset. Based on the algorithm of drug causality for Epidermal necrolysis (ALDEN), the Naranjo scale, and drug frequency (**Table 1**), Cadonilimab is considered the culprit, although the possibility of combined action with Lenvatinib cannot be completely ruled out.

**Table 1:** Evaluation of the suspected drugs in this case.

Suspected Drugs	ALDEN	Naranjo	Frequency	Additional Information
Cadonilimab	3^a^	6	Q3W	None
Lenvatinib	2^b^	3	QD	No reports of Lenvatinib-induced TEN; only one case of Toripalimab-Lenvatinib combination reported
Celecoxib	-1	1	BID	This patient continued using celecoxib after the resolution of TEN-like reactions with no adverse reactions
Hepatoprotective agents^c^	-3	1	QD	No reports of inducing TEN
TACE-related drugs^d^	-4	1	once, 35 days before	No reports of inducing TEN; the patient underwent TACE again 3 months later without adverse reactions

^a:^ The decrease in Cadonilimab’s ALDEN may be due to its recent market release and limited clinical use.

^b:^ The increase in Lenvatinib’s ALDEN may be due to concurrent initiation and cessation with Cadonilimab.

^c:^ These drugs include Ademetionine 1,4-Butanedisulfonate, Sodium Tiopronin, and Magnesium Isoglycyrrhizinate.

^d:^ These drugs include Oxaliplatin, Epirubicin, and Lipiodol.

**Figure 3:**
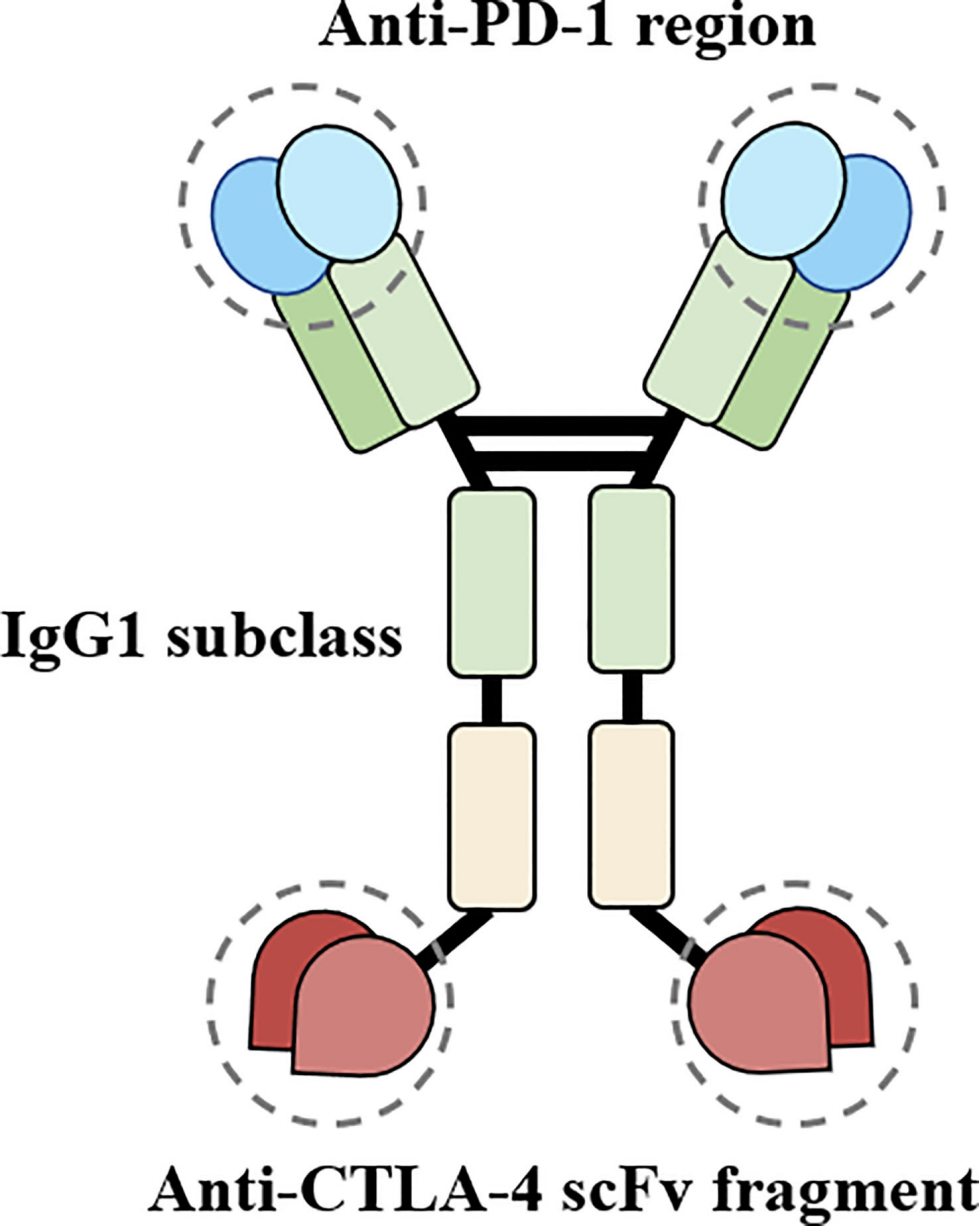
Simplified diagram of the tetravalent structure of Cadonilimab.

Cadonilimab blocks both PD-1 and CTLA-4 pathways (see **Figure 3** for the structural schematic (2)), thereby relieving their corresponding immunosuppressive effects and reversing tumor-specific T cell exhaustion. The efficacy of Cadonilimab for HCC was evaluated in a phase II clinical trial (NCT04444167) which revealed an objective response rate of 44.4% (8/18) and a disease control rate of 77.8% (14/18) while maintaining a manageable safety profile (4). In addition, Cadonilimab is an IgG1 scaffold Fc-engineered antibody modified to remove its binding to Fc receptors and C1q, preventing the antibody from mediating antibody-dependent cell-mediated cytotoxicity (ADCC), antibody-dependent cellular phagocytosis (ADCP), complement-dependent cytotoxicity (CDC), and antibody-dependent cytokine release in macrophages. Independent experiments have shown that compared to Cadonilimab in hG1WT format and other ICIs, Cadonilimab significantly downregulates Fc-mediated effector functions, including ADCC, ADCP, CDC, and does not activate macrophages to secrete interleukin-6 (IL-6) or IL-8, which are thought to be associated with irAEs (2). Current trials of Cadonilimab have not reported the occurrence of TEN. However, other ICIs have been reported to induce TEN, possibly due to overactivation of the immune system. Studies have shown that anti-PD-1 therapy increases the expression of its ligand, PD-L1, in keratinocytes, leading to cytotoxic CD8+ T cells promoting keratinocyte apoptosis, and ICIs-related eruptions have an analogous gene expression profile to TEN (5). Other possible mechanisms of irAEs include, but are not limited to, cross-reactivity between tumor cells and host tissues, off-target effects of ICIs on other target immune checkpoint ligands, disruption of self-tolerance leading to autoimmune reactivity and the production of pathogenic autoantibodies, and the abundance of certain microbial communities (6).

The critical step in managing SJS/TEN is to promptly discontinue the suspected drugs and initiate immunosuppressive treatment. According to version 5.0 of the National Cancer Institute Common Terminology Criteria for Adverse Events (NCICTCAE v5.0), TEN are classified as grade 4 irAEs. Therefore, the patient must permanently discontinue Cadonilimab. In the absence of prospective validation of the pathological and physiological mechanisms and preventive interventions related to Cadonilimab-induced TEN, the natural tendency is to compare it with other ICIs-related high-grade irAEs. A study reported the successful treatment of an HCC patient who experienced severe infusion-related reactions to Nivolumab (anti-PD-1) by using Pembrolizumab (anti-PD-1) with steroid premedication (7). Additionally, groundbreaking studies conducted in mouse models have shown that prophylactic TNF blockade can alleviate or prevent irAEs induced by dual CTLA-4 and PD-1 immunotherapy. Clinical trials have also suggested that TNF inhibition may be a safe therapeutic option for advanced cancer (8). Therefore, in the absence of alternative treatment options, late-stage cancer patients who experience similar irAEs may consider trying other ICIs after careful risk-benefit assessment, with close clinical monitoring, and the use of prophylactic non-targeted or targeted immunosuppressive agents.

Our case study provides a valuable reference for managing ICIs-associated TEN, which exhibits limited response to corticosteroids and IVIG. Notably, Adalimumab provided promising outcomes in our case, despite being less commonly utilized than Infliximab and Etanercept for treating TEN. Our findings are consistent with recent reports that highlight ICIs-associated TEN’s tendency towards drug resistance against corticosteroids and IVIG, and suggest Adalimumab as a viable alternative (9). Experimental observations have shown that TNF-α levels in blister fluid from TEN patients are much higher than those in serum, and TNF-α is overexpressed in keratinocytes, potentially inducing keratinocyte apoptosis through the caspase cascade and Fas/Fas ligand interaction (10). Baseline circulating levels of TNF-α are elevated in irAEs (6). These findings provide evidence for the use of TNF-α inhibitors in the treatment of ICIs-related TEN. Furthermore, TNF-α inhibitors may exert agonistic effects on cancer immune surveillance by preventing PD-L1 and T-cell Immunoglobulin and Mucin domain-containing protein-3 expression, as well as activation-induced cell death in CD8+ tumor-infiltrating lymphocytes (11). However, maintaining the balance of the immune activation spectrum is complex and critical, as it achieves intact cancer immune surveillance while inhibiting off-target autoimmunity. Therefore, a multidisciplinary evaluation is required to determine the potential impact of Adalimumab on specific cancer immune surveillance in the immunological context of TEN. Additionally, TNFR1 and TNFR2 control different responses and irAEs pathways. TNFR2 is preferentially expressed on highly immune-suppressive regulatory T cells and is associated with immune escape and tolerance. Therefore, selective TNFR2 inhibitors separate the anti-tumor effect of ICIs from autoimmune toxicity (12), providing a potential future treatment option for ICIs-related TEN. TNF-α inhibitors typically do not necessitate maintenance injections in cases of ICIs-related TEN, thus mitigating concerns regarding potential resistance, tuberculosis infection, or reactivation. Nonetheless, the use of immunomodulatory agents warrants prudence. It is imperative to ascertain the immunopathogenic mechanism of the disease, choose interventions that target key pathogenic drivers of specific toxicities, and take into consideration the organ-specific toxicity that may overlap with irAEs-affected sites.

Despite having the SCORTEN of 5, which predicts a mortality rate of 90%, this patient had a favorable outcome, in contrast to the poor prognosis observed in other cases (13) of ICIs-related TEN involving Ipilimumab and Nivolumab. This may be attributed to several factors, including Cadonilimab’s shorter terminal half-life (approximately 4.76 days (1), compared to Ipilimumab’s 14.7 days and Nivolumab’s 24.8 days, as reported in the Food and Drug Administration’s prescribing information), timely detection of irAEs, and supplementary treatment with Adalimumab.

The present case report has certain limitations. Given the lack of drug lymphocyte stimulation test, completely dismissing a particular scenario in which Cadonilimab indirectly induced irAEs is challenging. It is possible that Cadonilimab may have prompted an immunological response in the organism against previously tolerated concurrent medications, ultimately leading to TEN-like reactions. Further, it is difficult to accurately assess Adalimumab’s contribution to treatment due to its co-administration with other immunosuppressive agents. Additionally, because of the patient’s personal preference, there was no immunohistopathological analysis conducted on the affected tissues before the initiation of Adalimumab treatment. However, considering the obvious improvement in the patient’s condition following Adalimumab use, we retrospectively infer that TNF-α may be a key pathogenic driver of the specific toxicities observed in this Cadonilimab-related TEN-like reaction case. Further research is needed to determine the risk factors, pathogenesis, and optimal management strategies for ICIs-induced SJS/TEN. Therefore, personalized evaluation of patients’ immunopathogenesis (via immunohistochemistry, flow cytometry, multiplex cytokine analysis, and/or autoantibody measurements) (6) should be conducted whenever possible to supplement the dominant mechanism of injury and facilitate the development of targeted clinical decision-making.

Overall, this case report offers evidence of the effective resolution of Cadonilimab-related refractory TEN-like reactions in an immunotherapy-receiving HCC patient, with the addition of Adalimumab to routine treatment. These observations corroborate the notion that while Cadonilimab’s innovative structural design represents a daring breakthrough, its theoretical assertions regarding enhanced safety may require rigorous validation via a head-to-head clinical trial comparing registered ICIs. Despite ICIs having broadened the horizons of cancer therapy, the occurrence of high-grade irAEs manifested in this report and the over 90% prevalence of ICIs’ off-target side effects, as stated in The NCCN guidelines, reaffirms persistent concerns around the safety of ICIs-based immunotherapy. Therefore, it is crucial to remain vigilant and proactive in addressing these unresolved safety concerns surrounding ICIs therapy.

## Data availability statement

The original contributions presented in the study are included in the article/supplementary material. Further inquiries can be directed to the corresponding author.

## Ethics statement

The studies involving human participants were reviewed and approved by the ethics committee of Second Affiliated Hospital of Zhejiang University School of Medicine. The patients/participants provided their written informed consent to participate in this study. Written informed consent was obtained from the individual(s) for the publication of any potentially identifiable images or data included in this article.

## Author contributions

All authors were involved in the review of the data used for this case report, writing, editing and approving the manuscript for submission.
